# Risk Assessment of Importation and Local Transmission of COVID-19 in South Korea: Statistical Modeling Approach

**DOI:** 10.2196/26784

**Published:** 2021-06-01

**Authors:** Hyojung Lee, Yeahwon Kim, Eunsu Kim, Sunmi ‍Lee

**Affiliations:** 1 National Institute for Mathematical Sciences Daejeon Republic of Korea; 2 Kyung Hee University Yongin-si Republic of Korea

**Keywords:** COVID-19, transmission dynamics, South Korea, international travels, imported and local transmission, basic reproduction number, effective reproduction number, mitigation intervention strategies, risk, assessment, transmission, mitigation, strategy, travel, mobility, spread, intervention, diagnosis, monitoring, testing

## Abstract

**Background:**

Despite recent achievements in vaccines, antiviral drugs, and medical infrastructure, the emergence of COVID-19 has posed a serious threat to humans worldwide. Most countries are well connected on a global scale, making it nearly impossible to implement perfect and prompt mitigation strategies for infectious disease outbreaks. In particular, due to the explosive growth of international travel, the complex network of human mobility enabled the rapid spread of COVID-19 globally.

**Objective:**

South Korea was one of the earliest countries to be affected by COVID-19. In the absence of vaccines and treatments, South Korea has implemented and maintained stringent interventions, such as large-scale epidemiological investigations, rapid diagnosis, social distancing, and prompt clinical classification of severely ill patients with appropriate medical measures. In particular, South Korea has implemented effective airport screenings and quarantine measures. In this study, we aimed to assess the country-specific importation risk of COVID-19 and investigate its impact on the local transmission of COVID-19.

**Methods:**

The country-specific importation risk of COVID-19 in South Korea was assessed. We investigated the relationships between country-specific imported cases, passenger numbers, and the severity of country-specific COVID-19 prevalence from January to October 2020. We assessed the country-specific risk by incorporating country-specific information. A renewal mathematical model was employed, considering both imported and local cases of COVID-19 in South Korea. Furthermore, we estimated the basic and effective reproduction numbers.

**Results:**

The risk of importation from China was highest between January and February 2020, while that from North America (the United States and Canada) was high from April to October 2020. The R_0_ was estimated at 1.87 (95% CI 1.47-2.34), using the rate of α=0.07 for secondary transmission caused by imported cases. The R_t_ was estimated in South Korea and in both Seoul and Gyeonggi.

**Conclusions:**

A statistical model accounting for imported and locally transmitted cases was employed to estimate R_0_ and R_t_. Our results indicated that the prompt implementation of airport screening measures (contact tracing with case isolation and quarantine) successfully reduced local transmission caused by imported cases despite passengers arriving from high-risk countries throughout the year. Moreover, various mitigation interventions, including social distancing and travel restrictions within South Korea, have been effectively implemented to reduce the spread of local cases in South Korea.

## Introduction

The COVID-19 outbreak has affected people worldwide. A novel virus named SARS-CoV-2 was identified as the pathogen responsible for the outbreak of COVID-19 [1]. The common symptoms of COVID-19 include fever, dry cough, fatigue, chills, headache, and sore throat. Furthermore, severe symptoms of COVID-19, including high fever, severe cough, and shortness of breath, are often indicative of pneumonia [2]. The first case of COVID-19 was reported in Wuhan, China, in early December 2019. On March 11, 2020, the World Health Organization (WHO) declared the outbreak a global pandemic [3]. As of October 31, 2020, a total of 45,551,965 confirmed cases and more than 1,189,306 deaths were reported in 214 countries worldwide. The world has experienced a couple of epidemics caused by coronaviruses from the same family as SARS-CoV-2, such as severe acute respiratory syndrome (SARS) caused by SARS-CoV in 2003 and Middle East respiratory syndrome (MERS) caused by MERS-CoV in 2012, which had a large impact similar to that of the currently ongoing COVID-19 pandemic. However, the impact of COVID-19 is different in many aspects and has been more devastating than that of the other two outbreaks [4-6]. Due to the substantial growth of international travel, the complex network of human mobility has allowed pathogens to spread globally within a short time scale. In particular, the recent new COVID-19 variants have motivated the implementation of strengthened border control and lockdowns worldwide [7].

In this regard, most researchers have confirmed that COVID-19 was exported via air travel from mainland China. Researchers have developed many mathematical, statistical, and computational models to analyze air traffic data and estimate the consequent effects. Many researchers have investigated COVID-19 transmission dynamics in various ways. They have analyzed the characteristics of pathogen transmission cases in various experiments using elaborate computational models; their findings have enlightened us on how COVID-19 may affect us in the future. International air travel volume has been significantly related to the spread of COVID-19 worldwide. A network-driven model of global spread employed air traffic data to demonstrate and compare the impacts of the H1N1 epidemic in 2009 and the SARS epidemic in 2003 [8]. Furthermore, the risk of MERS-CoV exportation worldwide was evaluated by incorporating seasonal air traffic flows and the time-varying incidence of cases in Middle Eastern countries [9].

Various studies have investigated the global spread of COVID-19 during the early stages of the pandemic. One study examined how COVID-19 was imported into Europe by analyzing air traffic data [10]. Another study investigated the risk of transmission of COVID-19 through flights from four major cities in China (Wuhan, Beijing, Shanghai, and Guangzhou) to the passengers’ destination countries [11]. The study identified a risk index of COVID-19 transmission based on the number of travelers to destination countries, weighted by the number of confirmed cases in the departed city as reported by the WHO. The importation risk of COVID-19 cases by air travel from infected areas in China was assessed [12]. The risk before and after the travel ban in Hubei province was compared. Travel restrictions and border control measures have been enforced in China and other countries to limit the spread of the disease [13]. The results of a previous study showed that the daily risk of exporting a minimum of one COVID-19 case from mainland China via international travel exceeded 95% on January 13, 2020 [13].

Furthermore, the risk of imported COVID-19 cases was investigated in China by measuring a risk index from inbound international flights in previous studies [14,15]. These studies evaluated policy implications based on the index to adjust international air travel restrictions dynamically. Another study analyzed imported cases of COVID-19 in Taiwan in terms of characteristics, infection source, symptom presentation, and route of identification of imported cases [16]. The study confirmed that the strict enforcement of countermeasures was effective in preventing community transmission. The risks of both importation and exportation of COVID-19 have been investigated in a previous study [17]. The study evaluated the risk of importation and exportation of COVID-19 in all airports of 73 countries during the early stages of the pandemic until March 3, 2020.

The complex network of human mobility has been identified as an essential factor responsible for the rapid spread of COVID-19 globally. Due to the special situation between South Korea and North Korea, international flights are the most common way to enter South Korea. In particular, due to a large number of international travelers from China, South Korea was one of the earliest countries to experience a COVID-19 outbreak. In the absence of vaccines and treatments, South Korea implemented and maintained stringent interventions such as large-scale epidemiological investigations, rapid diagnosis, case isolation, contact tracing, quarantine, and social distancing. Despite the overall dramatic decrease in international flights, there is still a constant inflow of flights from high-risk countries. Therefore, the risk of COVID-19 in South Korea must be assessed.

In this study, we investigated the impact of international travel on the local transmission dynamics of COVID-19 in South Korea. First, we identified the relationship between the number of international travelers and country-specific confirmed cases of COVID-19. We computed the country-specific importation risk of COVID-19, accounting for the number of travelers entering South Korea, the number of confirmed cases in the originating countries, and the population of the originating countries. Second, statistical modeling was employed to capture the impact of secondary transmission caused by both imported and local cases of COVID-19 and determine the basic reproduction number (*R*_0_) and the effective reproduction number (*R*_t_). Finally, we assessed the impact of imported cases on local transmission of COVID-19.

## Methods

### Epidemiological Data

We analyzed country-specific epidemiological data on COVID-19 cases and international travel volume in South Korea from January to October 2020. First, data on the number of confirmed COVID-19 cases in South Korea were extracted from the Korea Disease Control and Prevention Agency (publicly available data) [18]. The epidemiological data included the dates of confirmation, dates of symptom onset, and transmission classification (local transmission/imported cases) [19]. Second, data on the monthly number of passengers entering South Korea in 2019-2020 were gathered from Incheon International Airport (publicly available data) [20]. Third, data on the number of confirmed cases of COVID-19 from the countries of origin were collected from the WHO situation report [21] and countries’ populations were obtained from [22]. The country-specific data are presented in Table S1 and Figures S1-S5 in Multimedia Appendix 1.

### Epidemiological Characteristics of COVID-19 Transmission Dynamics in South Korea

**Figure 1:**
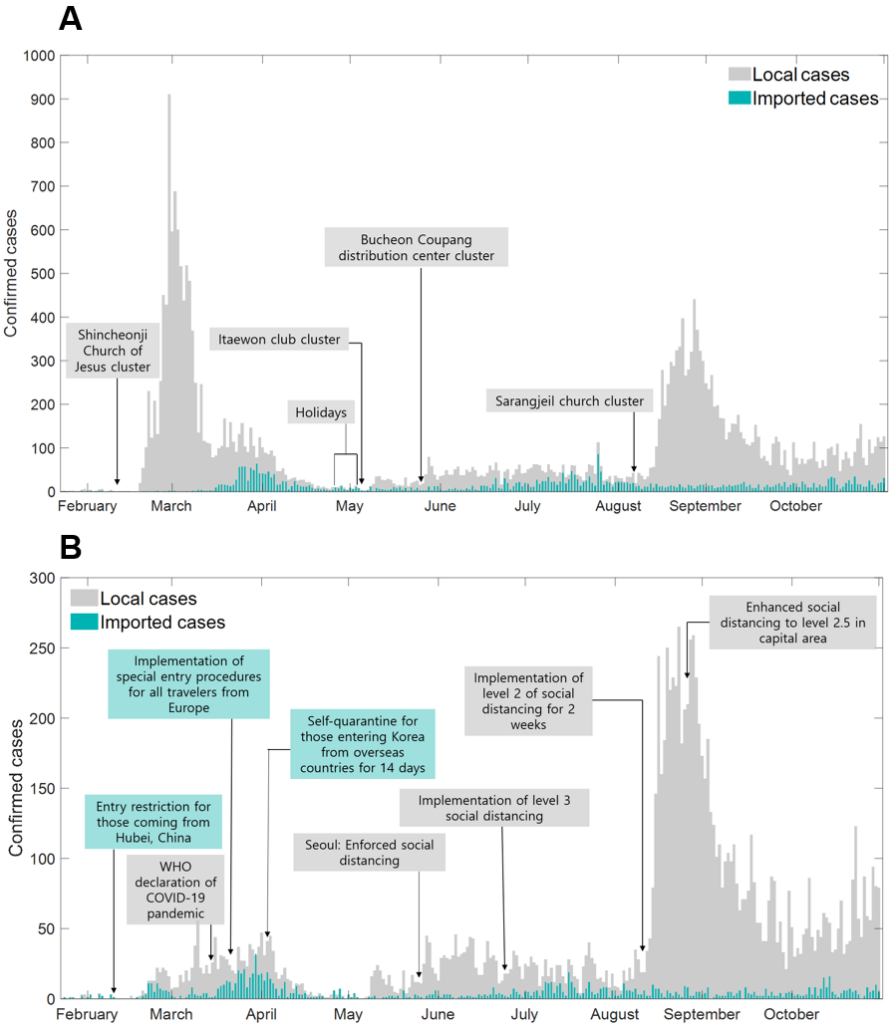
Epidemic curve of imported cases and local cases in South Korea. (A) Daily number of imported and local cases of COVID-19 in South Korea. (B) Daily number of imported and local cases in Seoul and Gyeonggi. Gray bars and green bars represent local cases and imported cases, respectively. WHO: World Health Organization.

We have presented the epidemic curves of local and imported cases in South Korea in Figure 1A and in Seoul and Gyeonggi in Figure 1B. Most COVID-19 cases occurred in March 2020 (due to the explosive outbreaks in Daegu and Gyeongbuk), and the number of cases in Seoul and Gyeonggi increased steadily from May 2020, leading to a larger outbreak in September 2020. Figure 1B shows the epidemic curve in Seoul and Gyeonggi along with the timeline of screening and quarantine interventions (green box) and social distancing interventions (gray box). The list of selected interventions is presented in Table S2 in Multimedia Appendix 1. The COVID-19 transmission dynamics in South Korea showed spatial heterogeneity. There were two major hotspots. First, the early outbreak was primarily in the Daegu and Gyeongbuk areas from February to April 2020 due to the Shincheonji Church–related clusters, as shown in Figure 1A and reported previously [23,24]. Second, the late outbreak was primarily in the Seoul and Gyeonggi areas in September and November 2020, as it was triggered by the Sarangjeil Church–related gathering on August 15, 2020, as shown in Figure 1A.

The timeline of the administrative measures implemented in South Korea is shown in Figure 1B and Table S2 in Multimedia Appendix 1. The Korean government, Seoul, and Gyeonggi implemented administrative countermeasures in response to the COVID-19 outbreak, including guidelines for entry restrictions followed by the 2-week self-quarantine guidelines from a different period combined with social distancing interventions.

### Risk of Importation of COVID-19

We aimed to calculate the country-specific importation risk of COVID-19 based on the number of international travelers, confirmed cases in the originating countries, and the population of the originating countries. The countries were grouped as Europe (the United Kingdom, Germany, and France); China and Asia except China; and North America (the United States and Canada). Country-specific importation risk is defined as a function of three factors: population, the number of confirmed cases of COVID-19, and passengers entering South Korea [25,26]. The risk of importation from a given country in a given month was derived as follows:

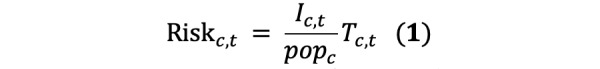



where *t* is the month from January 2020 to October 2020 (*t*=1,2,…,10) and *c* is a group of countries (*c*={China, Asia [except China], Europe, North America}). *I_c,t_* stands for the monthly confirmed cases of COVID-19 in a month *t* and an originating country *c*. The population-adjusted density of infectious travelers was obtained by *I_c,t_* dividing its population *pop_c_* of country *c*. *T_c,t_* represents the number of passengers traveling from country *c* in a month *t*. The normalized risk for country *c* in month *t* was as follows:

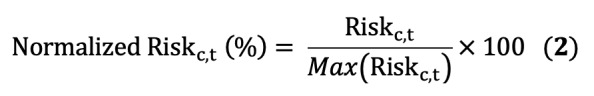



where *Max*(Risk_c,t_) indicates the maximum of the Risk_c,t_ for month *t* and country *c*. Moreover, we obtained a correlation between the monthly number of passengers and cases of COVID-19. We calculated the monthly Pearson correlation coefficients between the number of passengers and the number of COVID-19 cases corresponding to different countries including Japan, Vietnam, the Philippines, the United States, China, Thailand, Taiwan, Malaysia, Singapore, Germany, France, Canada, and the United Kingdom. The Pearson correlation coefficients are higher than 0.7 from April 2020, indicating that the number of passengers had a linear relationship with the number of COVID-19 cases in 2020. Overall, this implied that prompt country-specific surveillance should be implemented for a more cautious screening process that may be applied to passengers from higher-risk countries. The high correlation was due to two major countries—China was the highest risk country in the early stages of the pandemic, while the United States was the highest risk country in the later stages of the pandemic.

### Estimation of Reproduction Numbers

In this section, a renewal equation was employed to estimate the *R*_0_. The *R*_0_ is defined as the average number of susceptible individuals infected by a single primary case. Previous studies on COVID-19 estimated the *R*_0_ to be 2-3 [26-28]. In this study, we have categorized the total cases into locally transmitted (local cases) and imported cases. The total incidence of COVID-19 at time *t*, denoted by *i*(*t*), is the sum of local cases (*i_L_*(*t*)) and imported cases (*i_o_*(*t*))—that is, *i*(*t*)=*i_o_*(*t*)+*i_L_*(*t*). The renewal equation for the transmission dynamics of COVID-19 is defined as follows [29,30]:

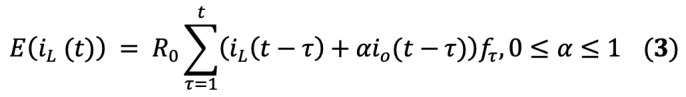



where *fτ* is the probability distribution of the serial interval in *τ*, and α is the relative contribution of the imported cases to secondary disease transmission (0≤α≤1) [27,31]. A serial interval is the time interval from illness onset in a primary case (infector) to that in a second case (infectee) [28]. The serial interval was assumed according to the gamma distribution with a mean of 4.8 days (SD 2.3 days) [31,32]. The likelihood function, assuming that the daily counts follow a Poisson distribution, is defined as the following:

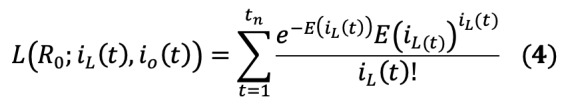



where *t_n_* is the final time. We estimated the *R*_0_ using the early confirmed cases from January 10 to February 25, 2020, using equations 3 and 4. Here, α is the relative contribution of imported cases to secondary disease transmission [27,31]. In Seoul and Gyeonggi, there were secondary confirmed cases of importation until April 2020, and there were very few secondary confirmed cases due to stringent interventions such as screening and the self-quarantine policy from April to June 2020. In total, 48 secondary cases related to imported cases were reported until June 2020, and the parameter α was calculated based on the total confirmed cases by April and June 2020. As of April 2020, the value of α was 7.57%, which was reduced to 3.63% in June 2020.

The *R*_0_ is relevant only in a largely susceptible population. Therefore, we also introduced the time-dependent reproduction number *R*_t_, calculated as the ratio of the number of new locally infected cases at time *t* and all infected individuals at time *t*. The details of the *R*_t_ computation can be found elsewhere [30,33]. The effective reproduction number was estimated on sliding windows of width *W* days, which was assumed to be a constant value over the time window (*W*-day average of *R*_t_).

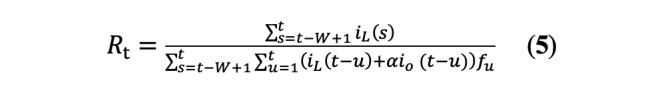



If *W*=1, the *R*_t_ is derived as follows:

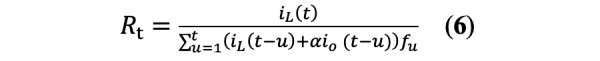



### Ethical Considerations

The data are presented in Table S1 in Multimedia Appendix 1. The data sets were fully anonymized and did not include any personally identifiable information. Thus, ethical approval was not required for the analysis.

## Results

### Overview

Relation between the number of passengers and imported cases was explored and the normalized country-specific risk was obtained. Among four groups of countries, China had a high risk of importation until February 2020. Afterward, North America showed a high risk of importation. The number of imported cases in Korea had a high correlation with the normalized risk, and the Spearman correlation coefficient was 0.82. The *R*_0_ was estimated at 1.87 (95% CI 1.47-2.34) with the rate of α=0.07 in Seoul and Gyeonggi. *R*_0_ was varied according to α to be between 1.83 and 3.94 in South Korea. The *R*_t_ in South Korea and in Seoul and Gyeonggi were shown and interpreted along with the control interventions.

### Imported and Local Cases of COVID-19 in South Korea

Figure S1 in Multimedia Appendix 1 illustrates a summary of the epidemiological data on COVID-19 cases in South Korea. The top panels of Figure S1A in Multimedia Appendix 1 show the overall characteristics of the confirmed cases from February to October 2020. The leftmost panel shows the ratio of COVID-19 cases by region: 43% in the Seoul and Gyeonggi areas, 33% in the Daegu and Gyeongbuk areas, and 24% in the rest of South Korea. The second panel shows the ratio of imported cases (14%) to local cases (86%). The third panel shows the number of confirmed cases in Seoul and Gyeonggi and the ratio of imported cases (10%) to local cases (90%). The rightmost panel shows that 31% of imported cases were reported in Seoul and Gyeonggi. Seoul and Gyeonggi are the regions with the most inflow of foreigners in South Korea as they have major international airports (ie, Incheon International Airport and Gimpo International Airport).

Figure S1B in Multimedia Appendix 1 shows the monthly proportion of imported cases from five continents and the total proportion of imported cases (top left panel). A list of country-specific imported cases is given in Table S1 in Multimedia Appendix 1. The number of imported cases from China was large during January and April 2020, while that from North America and Europe increased until April 2020. However, the number of imported cases from Asia increased rapidly from May to October 2020; Asia (50.6%), North America (28.1%), and Europe (17.8%) accounted for most of the cumulative imported cases.

### Relation Between the Number of Passengers and Imported Cases of COVID-19

**Figure 2:**
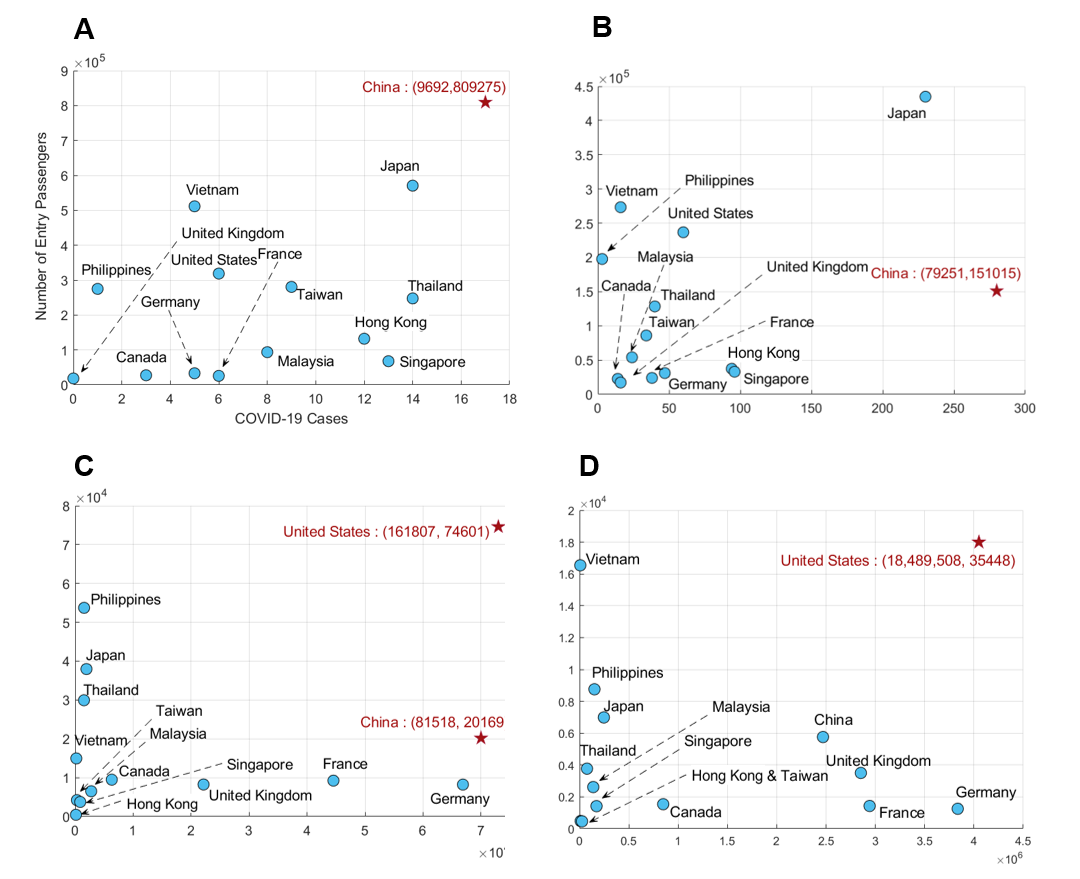
Relationship between the monthly number of passengers entering South Korea and COVID-19 cases at originating countries in (A) January, (B) February, (C) March, and (D) April 2020. The red stars represent particularly large numbers of COVID-19 cases (China and the United States).

The importation risk implies that a country with more COVID-19 cases and more travelers entering South Korea has a higher risk of importation. Figure 2 shows the relationship between the number of passengers entering South Korea in 2020 and the monthly confirmed cases of COVID-19 between January and April in 2020. After February 2020, the number of Chinese passengers rapidly decreased due to the emerging outbreak of COVID-19 in China, as shown in Figures 2A and 2B. The number of confirmed cases and the number of passengers entering South Korea from the United States increased from March to October 2020, as shown in Figure S2 in Multimedia Appendix 1. This indicated that the number of passengers was dramatically reduced since the COVID-19 pandemic began, owing to travel bans and restrictions (see Table S2 in Multimedia Appendix 1). Next, the number of international travelers who arrived in South Korea in 2020 was compared with that in 2019 (Figure S3 in Multimedia Appendix 1). The number of country-specific confirmed cases and passengers per month from the top 13 countries from January to October 2020 are shown in Figures S4 and S5 in Multimedia Appendix 1, respectively. The number of cases in China was reduced dramatically from March 2020 (Figure S4A in Multimedia Appendix 1), while the number of cases in the United States, the United Kingdom, France, Germany, Canada, and Malaysia continued to increase until October 2020 (Figures S4H-M in Multimedia Appendix 1). However, the number of passengers was greatly reduced, regardless of country, as shown in Figure S5 in Multimedia Appendix 1.

### Importation Risk of COVID-19

**Figure 3:**
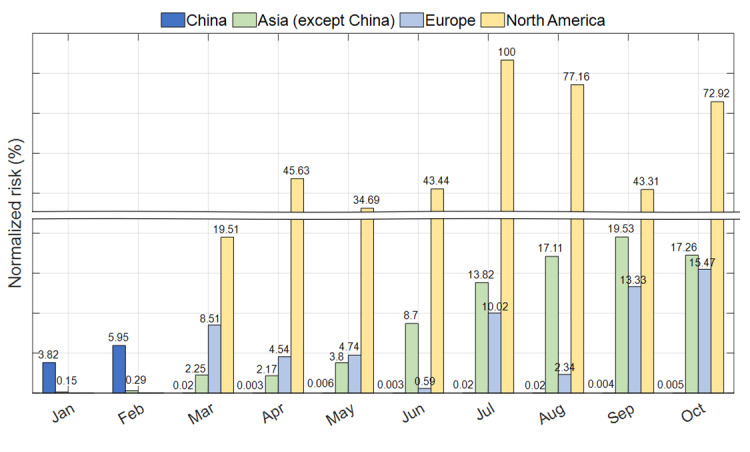
The country-specific risk of case importation from the top 13 countries to South Korea from January to October 2020.

We presented the risk of country-specific importation in Figure 3 and Table 1. Between January and February 2020, the risk of importation from China was the highest among the countries studied, while that from North America (the United States and Canada) showed a significantly high risk of importation from April to October 2020. The number of imported cases was highly correlated with the normalized risk (the Spearman correlation coefficient and Kendall correlation coefficient were 0.82 and 0.64, respectively).

**Table 1:** Normalized risk of importation in South Korea by country using data on population, number of COVID-19 cases, and number of passengers from January to October in 2020.

Country/region (population) and month		COVID-19 cases, n	Passengers, n	Normalized risk of importation, %
**Europe (214,921,407)**				
	January	11	77,183	0.002
	February	115	72,133	0.02
	March	141,116	25,656	8.51
	April	453,720	6182	4.54
	May	584,312	3450	4.74
	June	634,734	4963	0.59
	July	684,781	6224	10.02
	August	833,071	6701	2.34
	September	1,233,958	4595	13.33
	October	2,715,743	4441	15.47
**Asia, except China (3,032,800,000)**				
	January	168	5,535,607	0.15
	February	1254	1,397,128	0.29
	March	70,717	190,649	2.25
	April	224,609	57,968	2.17
	May	558,836	40,838	3.8
	June	1,105,224	47,219	8.7
	July	1,786,777	46,420	13.82
	August	2,709,219	37,908	17.11
	September	3,410,902	34,362	19.53
	October	3,009,642	34,419	17.26
**China (1,399,620,000)**				
	January	9701	1,089,779	3.82
	February	79,285	236,911	5.95
	March	81,824	24,381	0.02
	April	83,287	6242	0.003
	May	85,012	9479	0.006
	June	85,674	11,120	0.003
	July	88,423	15,323	0.02
	August	90,839	19,579	0.02
	September	91,480	18,690	0.004
	October	92,402	16,193	0.005
**North America (365,968,433)**				
	January	9	346,090	0.004
	February	74	259,247	0.02
	March	168,124	84,074	19.51
	April	1,061,615	36,987	45.63
	May	1,805,819	33,757	34.69
	June	2,640,886	37,676	43.44
	July	4,504,036	38,869	100
	August	5,982,879	37,783	77.16
	September	7,110,608	27,809	43.31
	October	8,989,268	28,109	72.92

**Figure 4:**
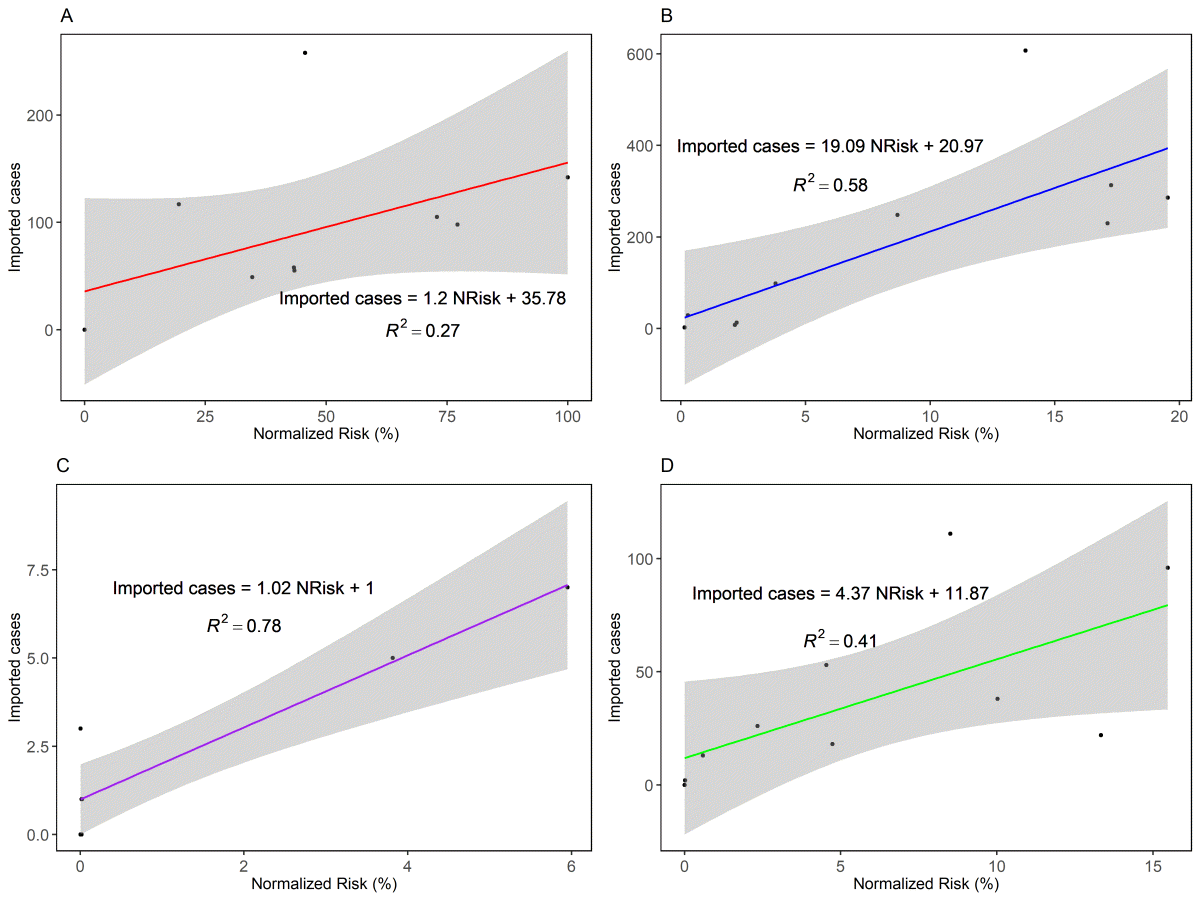
Regression analysis between the imported cases in South Korea from (A) North America, (B) Asia (except China), (C) China, and (D) Europe, and normalized risk (%). The dots indicate the number of imported cases in South Korea and solid lines represent the fitted linear regression. NRisk: normalized risk.

Figure 4 showed the results of regression analysis between the country-specific risk and the number of imported cases according to the four different countries/regions. The estimated values using the regression analysis are summarized in Table S3 in Multimedia Appendix 1. All countries had a positive relationship between imported cases and the risk of importation because the estimates of the slopes were positive. The linear regression models were well fitted, especially for China (*R*^2^=0.78). Asia (except China) was the most affected region with respect to the risk of importation. It is clear that imported cases entering from Asia (except China) can increase much more if the risk importation of Asia (except China) is elevated.

**Reproduction Number**

**Figure 5:**
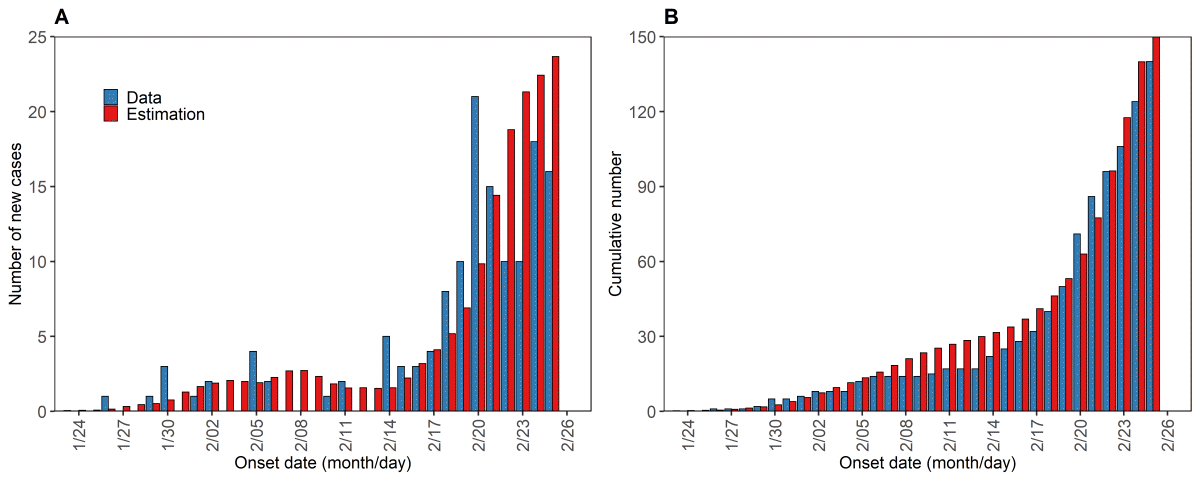
Comparison between estimated cases and observed cases of COVID-19 in Seoul and Gyeonggi using R_0_ when α=0.07. (A) Daily local cases in Seoul and Gyeonggi. (B) Cumulative local cases in Seoul and Gyeonggi. The red bar shows the estimated cases, while the blue bar shows the observed COVID-19 cases in the Seoul and Gyeonggi regions.

We varied α from 0 to 1 and estimated the *R*_0_ for the early COVID-19 outbreak in Seoul and Gyeonggi based on COVID-19 confirmed cases from January 10 to February 25, 2020, while the *R*_0_ in South Korea was based on COVID-19 confirmed cases from February 1 to February 19 (Table 2). Figure 5 shows the comparison between the COVID-19 data and estimated cases (Figure 5A) and cumulative local cases (Figure 5B). The corrected Akaike information criterion was calculated at 159.08 and the Bayesian information criterion was 160.84. Table 2 illustrates the estimation of *R*_0_ with varying α. The estimated *R*_0_ was 1.87 (95% CI 1.47-2.34) with α=0.07 and 1.49 (95% CI 1.17-1.87) with α=1.0. This indicated that the value of *R*_0_ decreased with increasing α since there was no secondary infection from imported cases (α=1).

**Table 2:** Estimation of R_0_ by α.

α value	*R*_0_ of Korea (95% CI)^a^	*R*_0_ of Seoul and Gyeonggi (95% CI)^a^
0.00	3.94 (2.18-6.04)	1.89 (1.49-2.38)
0.07	3.76 (2.11-5.75)	1.87 (1.47-2.34)
0.10	3.63 (2.05-5.58)	1.85 (1.46-2.33)
0.30	2.98 (1.71-4.66)	1.76 (1.38-2.21)
0.50	2.53 (1.47-4.00)	1.67 (1.31-2.10)
0.70	2.19 (1.28-3.50)	1.59 (1.25-2.00)
1.00	1.83 (1.08-2.94)	1.49 (1.17-1.87)

^a^95% CI was calculated from profile likelihood [34].

**Figure 6:**
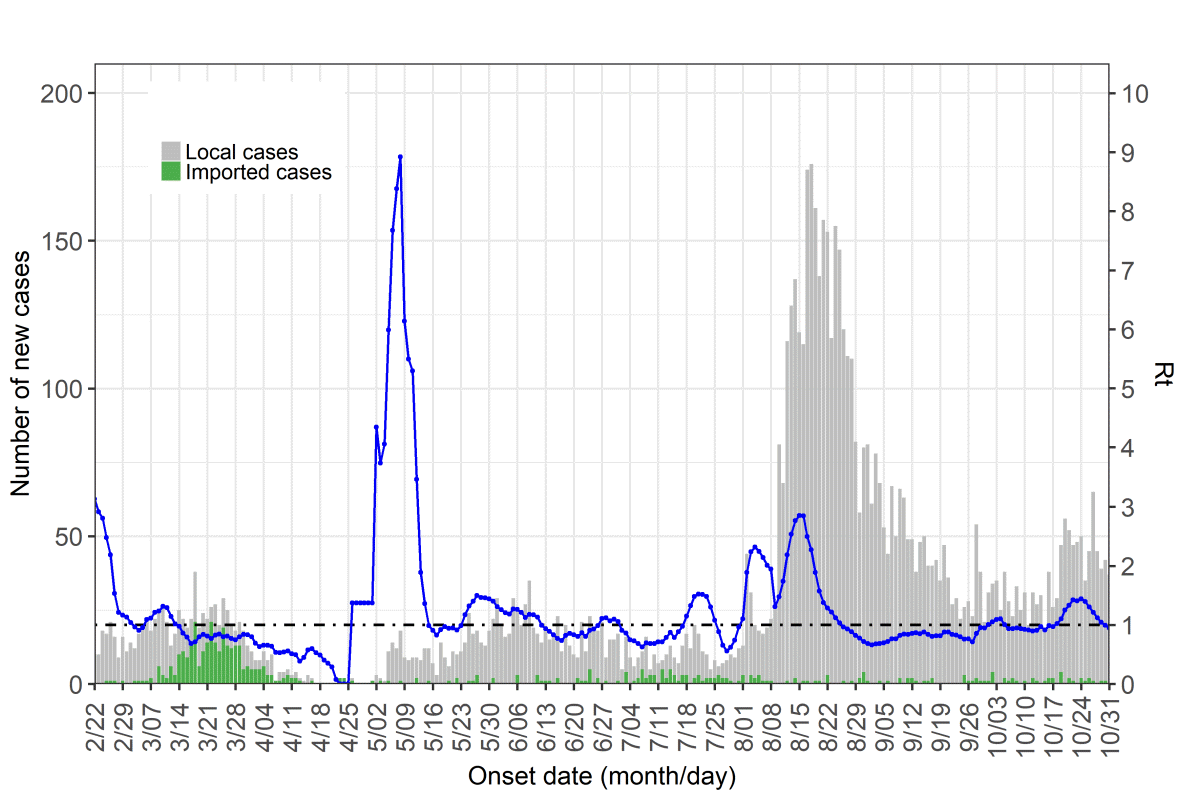
Local and imported cases and R_t_ in Seoul and Gyeonggi. The daily epidemic curves of imported (green) and local (gray) cases are shown. The blue curve shows the value of R_t_ when α=0.07 and the black horizontal line denotes R_t_=1.

The *R*_t_ was calculated for α=0.07 and the daily epidemic curves of imported (green) and local (gray) cases and the *R*_t_ values (blue curve) in the Seoul and Gyeonggi regions are shown in Figure 6. On March 11, 2020, when the WHO declared a pandemic situation, the *R*_t_ value fell below 1. At the beginning of the holiday season in early May 2020, the value again increased rapidly. In addition, there was a large outbreak in August and September 2020 due to a public gathering from all over South Korea on August 15, 2020. We conducted the sensitivity analysis for *R*_t_ according to the different time windows and α values (Figures S6A and S6B in Multimedia Appendix 1). Additionally, the *R*_t_ in South Korea from April to October 2020 is shown in Figures S6C and S6D in Multimedia Appendix 1.

## Discussion

### Principal Findings

In this study, we analyzed country-specific epidemiological data and data on passengers entering South Korea from January to October 2020. First, the correlation between the number of passengers and COVID-19 cases by country was calculated. A country with more confirmed cases showed a higher risk of importation. Second, the country-specific risk was highly correlated with the number of imported cases by country (Spearman correlation coefficient=0.82); China had the highest importation risk of COVID-19 in the early stages of the pandemic (January and February 2020), while North America (United States and Canada) showed the high importation risk from April to October 2020. Third, for the early stages of the COVID-19 pandemic, the *R*_0_ was estimated at 1.87 (95% CI 1.47-2.34), which was similar to the *R*_0_ of COVID-19 of approximately 2-3 in Wuhan, China [28,35,36]. Finally, we estimated the *R*_t_ by employing the renewal equation, accounting for the effects of control interventions.

Mainland China and South Korea experienced a steep rise in the number of COVID-19 confirmed cases in the early stages of the COVID-19 pandemic [3,32]. It appears that the two governments’ respective reactions to the novel virus resulted in a successful reduction in infection rates; however, the cost burden that the two countries had to bear was quite different. China implemented a lockdown in Hubei province and strict border control measures against higher-risk countries [37,38]. In China, active measures driven by the central government to retard the progress of epidemic diseases appear to be effective in impeding the spread of COVID-19; however, Chinese people had to pay burdensome costs during the initial outbreak of COVID-19 [39]. South Korea used strict social distancing measures and self-quarantine without restricting borders. However, South Korea expanded the volume of testing and promptly traced the contacts of confirmed cases [32,40].

There were potential risk factors that could have led to a larger outbreak of COVID-19 in South Korea. First, negative serial intervals indicated presymptomatic transmissions, highlighting the potential risk of transmission from asymptomatic cases. In South Korea, 12.7% of serial intervals were negative (199 pairs among 1567 symptomatic pairs), which could lead to large outbreaks as secondary transmission before the appearance of symptoms cannot be prevented [40,41]. Second, the *R*_0_ was estimated at 1.49 in Seoul and Gyeonggi, indicating that an epidemic might be possible. Third, the number of imported cases increased substantially from April 2020, although passenger volume has been rapidly decreasing due to the increase in COVID-19 cases. However, a large outbreak of COVID-19 caused by imported cases did not occur because of the policy of testing passengers arriving from other countries and isolating them for a minimum of 2 weeks; this policy was implemented on April 1, 2020, and reduced the risk of the spread of COVID-19.

This study has several limitations. First, this study relied on confirmed cases in South Korea. However, there were a substantial number of asymptomatic infections as 12.5% of serial intervals were negative [41], which represents presymptomatic transmission [32,40]. Thus, we did not consider secondary transmission caused by imported and local cases. Second, we analyzed the impact of imported cases on the local transmission of COVID-19 and found that the impact of imported cases had decreased since the strict implementation of airport screening and quarantine measures from April 2020 [24,42] (Table S2 in Multimedia Appendix 1). Therefore, the potential risk of infection from imported cases was regarded as a less important factor. However, the reason for the small outbreaks resulting from imported cases was due to strengthened screening inspection and self-quarantine measures for those entering South Korea, which were implemented on April 1, 2020. This means that secondary transmission by imported cases can play a critical role in COVID-19 transmission. Finally, the risk was estimated by month as monthly passenger volume data were available. If daily data were given, the risk by country could be computed daily or weekly. However, we observed a significantly different risk of importation of COVID-19 from overseas countries.

Despite these limitations, we investigated the risk of importation of COVID-19 using country-specific epidemiological data and passenger volume. By combining social distancing, screening, and self-quarantine for all travelers entering South Korea, the mitigation of COVID-19 transmission caused by imported cases in South Korea was highly successful. These efforts—accompanied by identification of the source of infection and strengthened quarantine measures for travelers from overseas countries—should be continued. Therefore, it is urgent to assess the risk of importation and maintain an effective surveillance system for COVID-19 in South Korea.

Strict control interventions were implemented to prevent the spread of COVID-19 in South Korea since the first case was confirmed in the country on January 20, 2020. The COVID-19 outbreak in South Korea has been successfully suppressed without strict lockdowns. First, the Korean government has constructed a rapid testing and diagnosis system [40,43]. Previous studies have shown that most cases have been confirmed within a week of illness onset [24,43]. Moreover, drive-through screening centers were initiated on February 23, 2020, in Daegu, South Korea [44]. This system contributed to the rapid diagnosis and further testing of suspected cases. The entire drive-through testing procedure takes about 10 minutes, and it is helpful for diagnosing infections early in cases with mild symptoms or asymptomatic cases. Second, the widespread epidemiological investigation of contact tracing was conducted in infected as well as suspected cases [40]. Social distancing strategies and mask wearing have been recommended since February 2020. Social distancing has been found to mitigate the spread of COVID-19 cases [40,45,46]. Third, the Korean government introduced a “special entry procedure,” which was applied to all passengers from mainland China from February 4, 2020, to control imported cases. Subsequently, all passengers from overseas countries were quarantined for 14 days after April 1, 2020. Combined control interventions, including social distancing efforts, appear to have succeeded in preventing the spread of COVID-19 in South Korea.

Since the severity of COVID-19 outbreaks and health policies differ across countries, a country-specific surveillance system would be more efficient than a uniform screening and surveillance policy for every country. Another notable feature of South Korean border control is that the number of international travel hubs is limited, and passenger traffic can be effectively monitored. This, in turn, helps in diagnosing and tracing imported cases without imposing a strict lockdown. It is important to estimate the country-specific risk of importation by identifying high-risk countries to prevent recurrent outbreaks due to importation of cases. It would be helpful to access a finer level of information to estimate the effective reproduction number as the risk indicator of importation and local transmission. Therefore, our framework can also be applied to countries that have similar immigration policies. Furthermore, a risk assessment of imported cases between neighboring countries that do not implement border control (eg, the Schengen zone or Central America-4 Free Mobility Agreement) might be challenging.

### Conclusions

Data on international passengers entering South Korea, the severity of the COVID-19 outbreak in originating countries, and country-specific imported cases were analyzed to compute the risk of importation of COVID-19 into South Korea. China was a high-risk country for importation in the early stages of the pandemic until March 2020, while the United States and Canada showed a high risk of importation after April 2020. Moreover, statistical model accounting was employed to estimate the *R*_0_ and *R*_t_ using epidemiological data on imported and locally transmitted cases. Our results highlighted that rapid diagnosis and prompt implementation of case isolation and quarantine were effective in preventing secondary infections caused by imported cases through the continuous inflow of passengers traveling from high-risk countries. Therefore, multiple mitigation interventions (social distancing, a rapid diagnosis system, and movement restriction) should be implemented to reduce the spread of local and imported cases in South Korea.

## Appendix

Multimedia Appendix 1 Supplementary data.

## Abbreviations

MERS: Middle East respiratory syndrome
SARS: severe acute respiratory syndrome
WHO: World Health Organization
