# Supplementary material for: Risk Assessment of Importation and Local Transmission of COVID-19 in South Korea: Statistical Modeling Approach
Source: JMIR Public Health Surveill. 2021 Jun 1;7(6):e26784. doi: 10.2196/26784 (PMC8171290; doi:10.2196/26784)

**Table S1.** Weekly local and imported cases of COVID-19 in South Korea from February to October in 2020

| **Week** | | **Local** | **Imported** | **Country-specific imported cases** | | | | |
| --- | --- | --- | --- | --- | --- | --- | --- | --- |
|  |  |  |  | China | Asia (except China) | Europe | America | Africa |
| Feb | Week 1 | 41.7% | 58.3% | 4(57.1) | 3(42.9) | 0 | 0 | 0 |
|  | Week 2 | 0% | 100% | 2(100.0) | 0 | 0 | 0 | 0 |
|  | Week 3 | 99.5% | 0.5% | 0 | 3(100.0) | 0 | 0 | 0 |
|  | Week 4 | 99.8% | 0.2% | 2(28.6) | 2(28.6) | 3(42.9) | 0 | 0 |
| Mar | Week 1 | 99.9% | 0.1% | 1(25.0) | 0 | 3(75.0) | 0 | 0 |
|  | Week 2 | 98.2% | 1.8% | 0 | 4(2.6) | 147(97.4) | 0 | 0 |
|  | Week 3 | 87.1% | 12.9% | 0 | 14(15.6) | 59(65.6) | 15(16.7) | 2(2.2) |
|  | Week 4 | 52.3% | 47.7% | 0 | 31(9.8) | 182(57.8) | 102(32.4) | 0 |
| Apr | Week 1 | 68.8% | 31.2% | 0 | 21(10.5) | 90(45.0) | 88(44.0) | 1(0.5) |
|  | Week 2 | 56.0% | 44.0% | 0 | 6(5.1) | 32(27.1) | 80(67.8) | 0 |
|  | Week 3 | 44.3% | 55.7% | 0 | 11(13.6) | 18(22.2) | 52(64.2) | 0 |
|  | Week 4 | 44.8% | 55.2% | 1(2.8) | 7(19.4) | 7(19.4) | 21(58.3) | 0 |
|  | Week 5 | 20% | 80% | 2(3.9) | 18(35.3) | 14(27.5) | 17(33.3) | 0 |
| May | Week 1 | 89.6% | 10.4% | 0 | 17(53.1) | 2(6.3) | 9(28.1) | 4(12.5) |
|  | Week 2 | 89.4% | 10.6% | 0 | 18(50.0) | 5(13.9) | 11(30.6) | 2(5.6) |
|  | Week 3 | 89.2% | 10.8% | 0 | 25(69.4) | 1(2.8) | 10(27.8) | 0 |
|  | Week 4 | 89.0% | 11.0% | 0 | 28(53.8) | 5(9.6) | 19(36.5) | 0 |
| Jun | Week 1 | 89.0% | 11.0% | 0 | 16(50.0) | 3(9.4) | 13(40.6) | 0 |
|  | Week 2 | 88.9% | 11.1% | 0 | 30(65.2) | 6(13.0) | 10(21.7) | 0 |
|  | Week 3 | 88.4% | 11.6% | 0 | 59(67.8) | 2(2.3) | 19(21.8) | 7(8.0) |
|  | Week 4 | 87.8% | 12.2% | 0 | 115(85.8) | 3(2.2) | 13(9.7) | 3(2.2) |
| Jul | Week 1 | 87.1% | 12.9% | 0 | 94(77.7) | 6(5.0) | 19(15.7) | 2(1.7) |
|  | Week 2 | 86.1% | 13.9% | 0 | 121(69.5) | 15(8.6) | 36(20.7) | 2(1.1) |
|  | Week 3 | 85.0% | 15.0% | 0 | 147(77.8) | 5(2.6) | 32(16.9) | 5(2.6) |
|  | Week 4 | 83.7% | 16.3% | 0 | 195(81.9) | 8(3.4) | 30(12.6) | 5(2.1) |
|  | Week 5 | 82.9% | 17.1% | 0 | 115(76.2) | 5(3.3) | 25(16.6) | 6(4.0) |
| Aug | Week 1 | 82.5% | 17.5% | 0 | 51(54.3) | 4(4.3) | 30(31.9) | 9(9.6) |
|  | Week 2 | 82.9% | 17.1% | 0 | 43(48.9) | 5(5.7) | 26(29.5) | 14(15.9) |
|  | Week 3 | 84.5% | 15.5% | 0 | 46(57.5) | 11(13.8) | 16(20.0) | 7(8.8) |
|  | Week 4 | 85.8% | 14.2% | 1(1.1) | 51(58.6) | 5(5.7) | 26(29.9) | 4(4.6) |
| Sep | Week 1 | 86.4% | 13.6% | 3(4.0) | 39(52.0) | 8(10.7) | 18(24.0) | 7(9.3) |
|  | Week 2 | 86.5% | 13.5% | 0 | 99(88.4) | 1(0.9) | 9(8.0) | 3(2.7) |
|  | Week 3 | 86.6% | 13.4% | 0 | 47(60.3) | 12(15.4) | 18(23.1) | 1(1.3) |
|  | Week 4 | 86.5% | 13.5% | 0 | 83(82.2) | 5(5.0) | 13(12.9) | 0 |
| Oct | Week 1 | 86.4% | 13.6% | 0 | 69(67.6) | 18(17.6) | 11(10.8) | 4(3.9) |
|  | Week 2 | 86.2% | 13.8% | 1(1.0) | 68(65.4) | 15(14.4) | 14(13.5) | 6(5.8) |
|  | Week 3 | 86.0% | 14.0% | 0 | 80(64.5) | 16(12.9) | 26(21.0) | 2(1.6) |
|  | Week 4 | 85.9% | 14.1% | 2(1.5) | 64(49.2) | 33(25.4) | 25(19.2) | 6(4.6) |
|  | Week 5 | 85.8% | 14.2% | 0 | 66(58.4) | 17(15.0) | 28(24.8) | 2(1.8) |

Parenthesis refer to the percentage.

**Table S2.** Administrative quarantine measures in South Korea from February to August 2020

|  | **Administrative countermeasures** | **Ref** |
| --- | --- | --- |
| **February 4** | Entry restriction for those coming from Hubei, China | KCDC COVID-19 Response [23] |
| **February 23** | The alert level was raised to level 3 | KCDC COVID-19 Response [23] |
| **February 29** | Entry restriction for those coming from Korea in 76 countries | KCDC COVID-19 Response [23] |
| **March 11** | WHO declaration of global COVID-19 pandemic | [24] |
| **March 16** | Implementation of special entry procedures* to all travelers from Europe | KCDC COVID-19 Response [23] |
| **March 19** | Implementation of special entry procedure for all Koreans and foreigners entering South Korea | KCDC COVID-19 Response [23] |
| **April 1** | Self-quarantine for those entering Korea from overseas countries for 14 days | KCDC COVID-19 Response [23] |
| **May 29** | Enforced social distancing (Seoul, May 29-June 14) | KCDC Public Advice & Notice [25] |
| **June 28** | Implementation of social distancing of three-level | KCDC Public Advice & Notice [25] |
| **August 16** | Implementation of level 2 of social distancing for 2 weeks from 16 August | KCDC Guidelines[26] |
| **August 30** | Enhanced social distancing to level 2.5 in the capital area including Seoul, Gyeonggi, and Incheon | KCDC [27] |

Special entry procedures* implemented for travelers from China, Japan, Iran, and European countries. It was expanded to all Koreans and foreigners on March 19, 2020.

**Table S3.** Regression Analysis. The imported cases in South Korea according to the country was fitted with the linear regression (i.e., Imported cases = $\beta\times Risk+\alpha$ )

|  | Parameters | Estimates | 95% CI | *P*-value | $R^{2}$ |
| --- | --- | --- | --- | --- | --- |
| North America | $\alpha$ | 35.78 | (-51.17, 122.74) | 0.37 | 0.27 |
|  | $\beta$ | 1.20 | (-0.42,2.82) | 0.13 |  |
| Asia  (except China) | $\alpha$ | 20.97 | (-126.92, 168.86) | 0.75 | 0.58 |
|  | $\beta$ | 19.09* | (5.93, 32.25) | <0.05 |  |
| China | $\alpha$ | 1.00 | (-0.0007, 1.99) | 0.05 | 0.78 |
|  | $\beta$ | 1.02* | (0.57, 1.47) | <0.05 |  |
| Europe | $\alpha$ | 11.88 | (-21.96, 45.71) | 0.44 | 0.41 |
|  | $\beta$ | 4.37* | (0.14, 8.60) | <0.05 |  |

The symbol, * , indicates the statistically significant with the significant level 5%. 95% CI represents the 95% confidence interval.

**Figure S1.** Summary of COVID-19 confirmed cases from January to October 2020 in South Korea: **A.** Ratio of COVID-19 confirmed cases by region in South Korea, and the ratio of imported cases to local transmission cases. **B.** Monthly imported cases of COVID-19 from five continents.


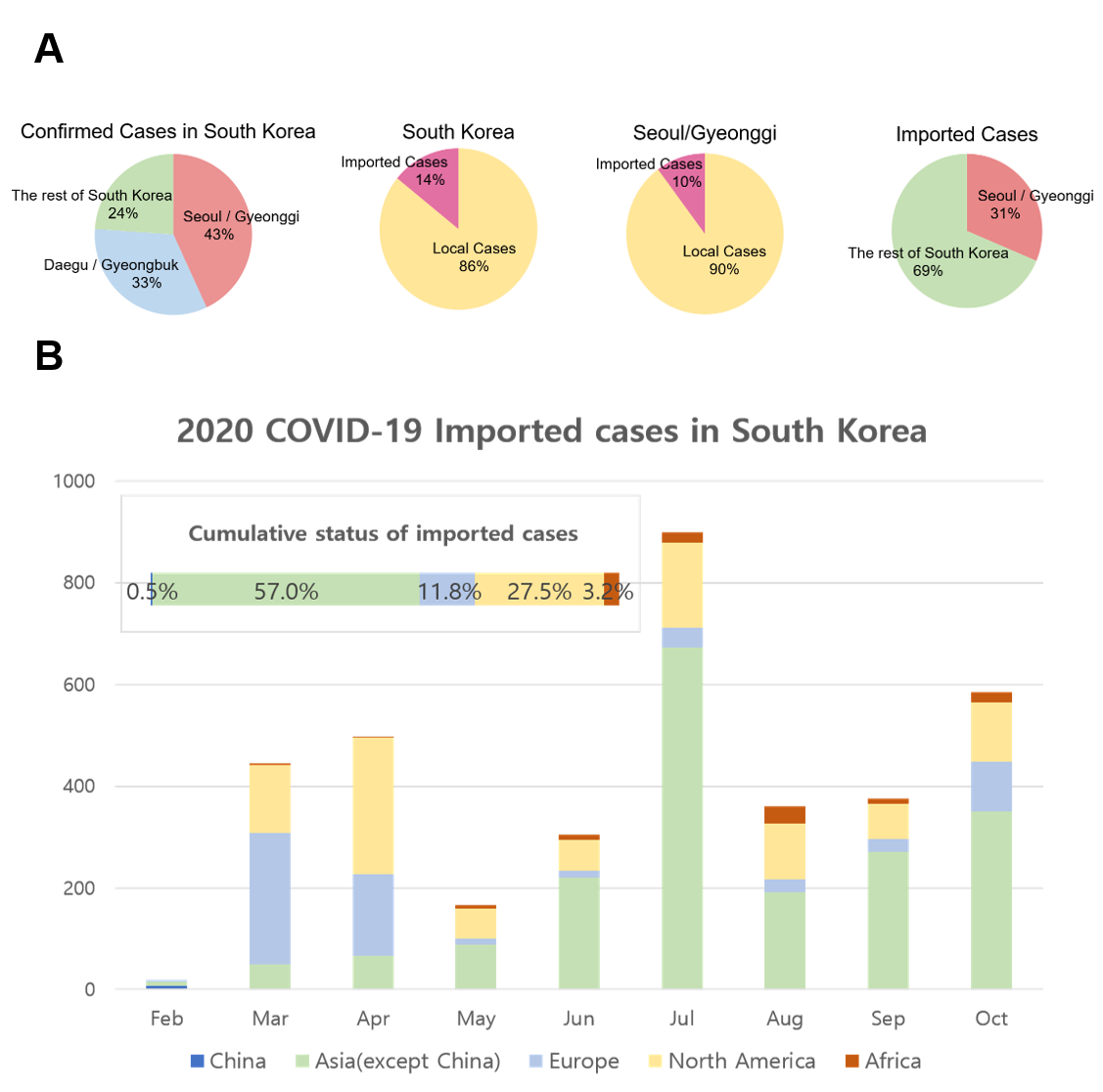


**Figure S2.** Relationship between the monthly number of passengers entering South Korea and COVID-19 cases at originating countries in (A) May, (B) June, (C) July, (D) August, (E) September, (F) October. The red star represents the substantially large number of COVID-19 cases (the United States).


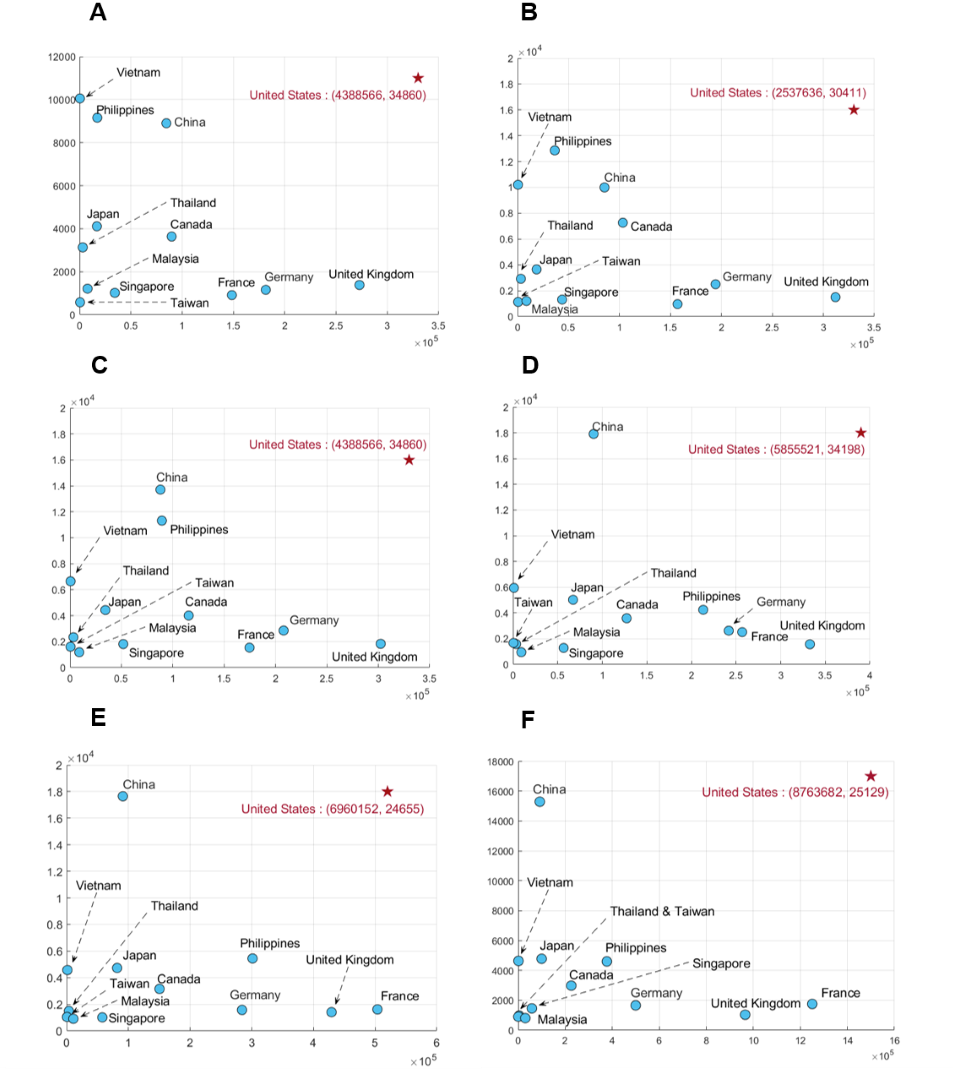


**Figure S3.** Comparison of the monthly number of passengers entering South Korea in 2019 and 2020. Dramatic reduction in 2020 is shown due to worldwide border control for COVID-19.


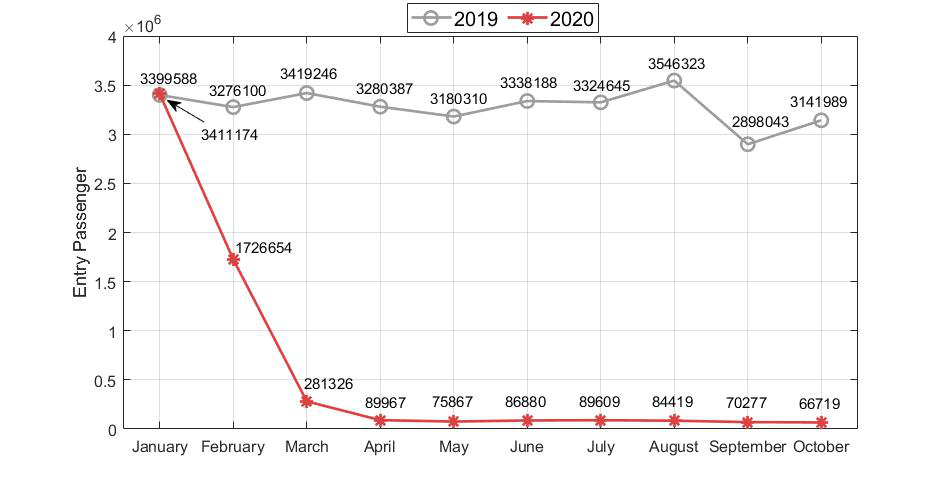


**Figure S4.** Comparison of the number of passengers entering South Korea from the top 13 countries between January and October 2020. The bar graph shows the monthly number of passengers (left vertical axis) and the solid curve shows the cumulative number of passengers (the right vertical axis).


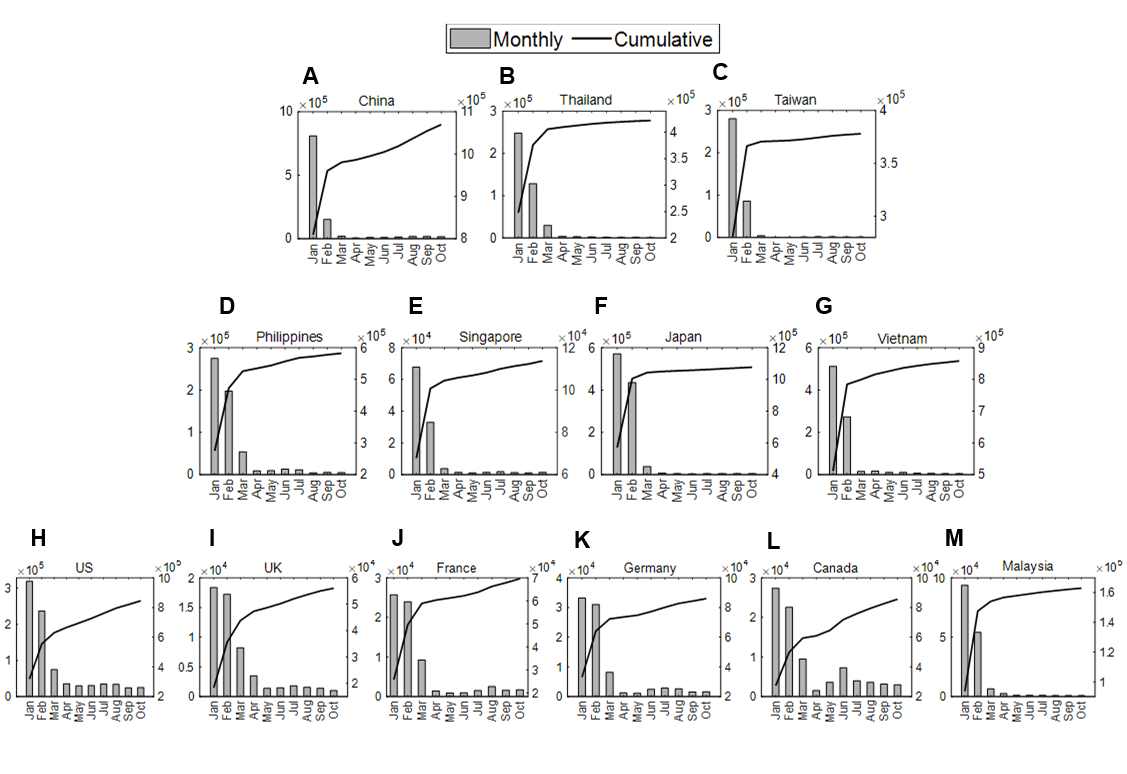


**Figure S5.** Comparison of the number of COVID-19 cases in the top 13 originating countries from January to October 2020. The bar graph shows the monthly number of COVID-19 cases (left vertical axis), and the solid curve shows the cumulative number of COVID-19 cases (the right vertical axis).


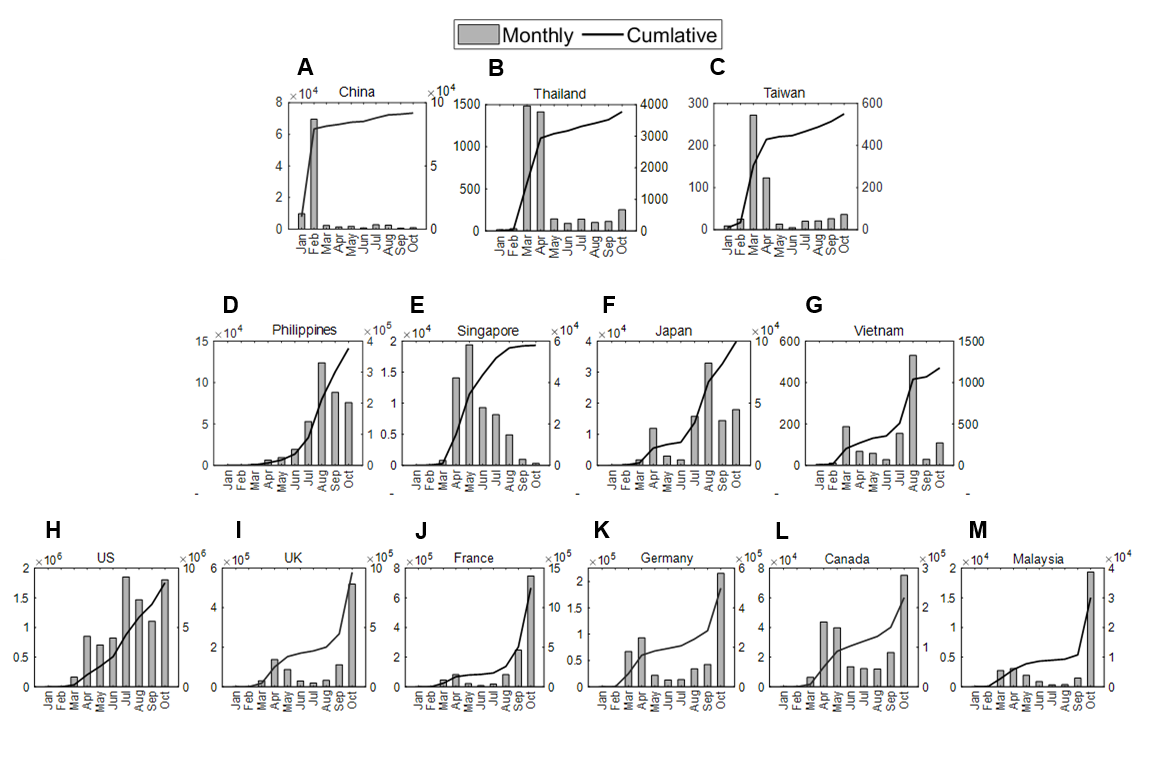


**Figure S6.** Effective reproduction number as varying the reduction rate of secondary transmission caused by imported cases ($\alpha$) and time window (W). The gray bars show local cases, the green bars show imported cases, while the blue curve shows the value of $R_{t}$. The black horizontal line shows $R_{t}=1$. **A**. Daily effective reproduction number in Seoul and Gyeonggi using $\alpha=1, W=1$. **B**. daily effective reproduction number in Seoul and Gyeonggi using $\alpha=1, W=7$ in Seoul and Gyeonggi. **C**. Daily effective reproduction number in South Korea using $\alpha=1, W=1$. **D**. The daily effective reproduction number in South Korea using $\alpha=1, W=7$.


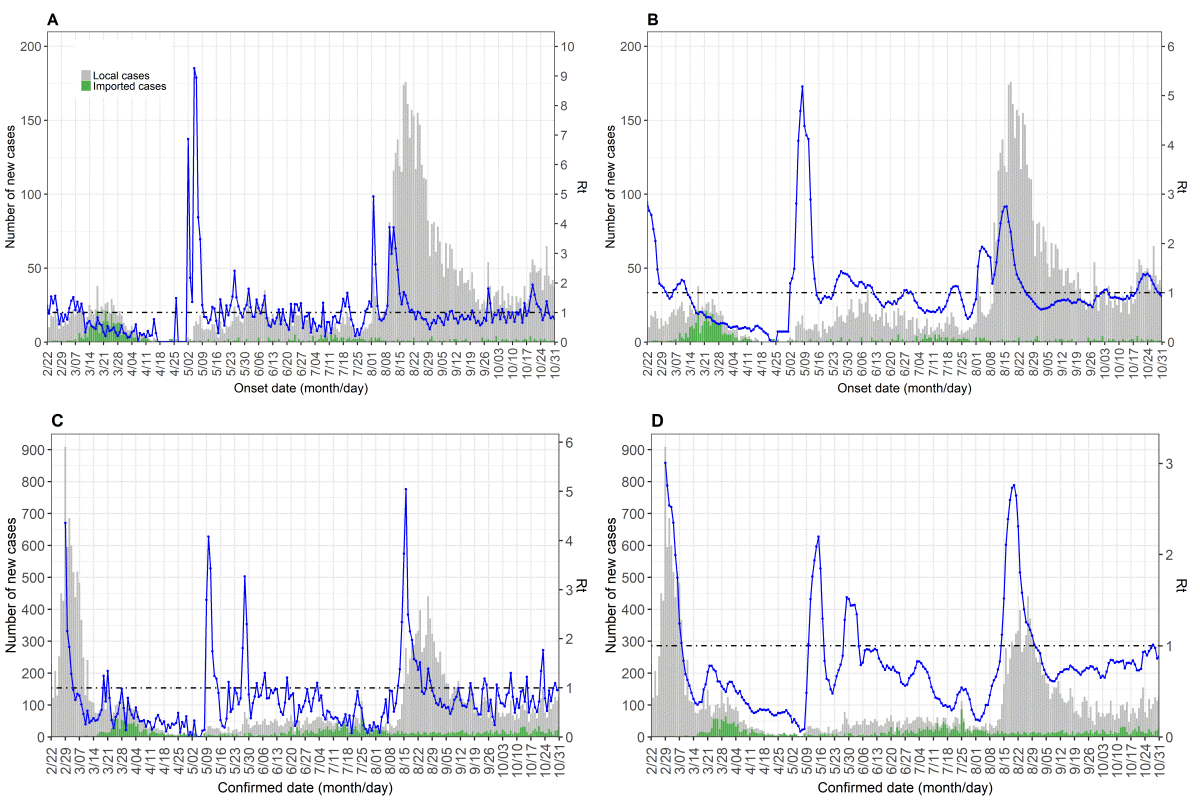

Supplement: Multimedia Appendix 1 [file publichealth_v7i6e26784_app1.docx]
